# Hyaluronan Signaling Ameliorates the Epithelial Injury Response and Barrier Disruption After Ozone Exposure

**DOI:** 10.3390/biom16060795

**Published:** 2026-05-28

**Authors:** Jonas Ritter, Vandy P. Stober, Carol S. Trempus, Yosuke Sakamachi, Jian-Liang Li, Erica L. Scappini, Anastasiya Birukova, Mohamed A. Elaguech, Adam R. Hall, Robert M. Tighe, Oliver H. Wittekindt, Stavros Garantziotis

**Affiliations:** 1 Division of Intramural Research, National Institute of Environmental Health Sciences, Research Triangle Park, Durham, NC 27709, USA; jonas.ritter.01@gmx.de (J.R.); parron@niehs.nih.gov (V.P.S.); trempus@niehs.nih.gov (C.S.T.); yosuke.sakamachi@nih.gov (Y.S.); jianliang.li@nih.gov (J.-L.L.); scappinie@niehs.nih.gov (E.L.S.); 2 Institute of General Physiology, Ulm University, 89081 Ulm, Germany; oliver.wittekindt@uni-ulm.de; 3 Department of Medicine, Duke University Medical Center, Durham, NC 27710, USA; abirukova@unither.com (A.B.); robert.tighe@duke.edu (R.M.T.); 4 Wake Forest School of Medicine, Virginia Tech-Wake Forest University School of Biomedical Engineering and Sciences, Winston-Salem, NC 27157, USA; mohamed.elaguech@advocatehealth.org (M.A.E.); arhall@wakehealth.edu (A.R.H.)

**Keywords:** ozone, airway epithelial cells, hyaluronan, Cd44, Rhamm, Tlr5

## Abstract

Airway pollutants, like the reactive oxygen species ozone, cause significant lung injury, which can lead to the development or exacerbation of lung diseases like asthma and chronic obstructive pulmonary disease (COPD), and drives worldwide morbidity and mortality. Altered epithelial function is a hallmark and trigger of ozone-induced lung injury, but its precise mechanisms are incompletely known. The extracellular matrix, and specifically its major component, hyaluronan (HA), plays a crucial role in cellular injury responses. We hypothesized that HA signaling mediates epithelial responses to ozone-induced injury. We exposed human and murine differentiated primary epithelia to ozone in vitro and evaluated epithelial integrity and transcriptomic responses. We used genetically deficient cells for cognate HA receptors cluster of differentiation 44 (CD44) and receptor for HA-mediated motility (RHAMM), and innate immune receptors toll-like receptor 4 (TLR4) and TLR5 to study signaling pathways, and evaluated high molecular weight HA (HMWHA) as a potential treatment for ozone-induced epithelial injury. In vitro ozone exposure caused significant reduction in epithelial integrity and very similar inflammatory changes in human and murine cells. CD44 deficiency led to decreased inflammation, while RHAMM deficiency exacerbated cell injury. HMWHA protected against ozone-induced epithelial injury, mediated by TLR4 and TLR5 but not CD44 or RHAMM. Our results identify novel contributions of HA signaling to ozone-induced epithelial injury and suggest that HMWHA protects epithelia via innate immune activation of TLR4 and TLR5.

## 1. Introduction

The environment plays a pivotal role in human health. This is especially evident in the lung, which is constantly exposed to the ambient environment. It is estimated that humans inhale 11,000 L of air daily [1], which exposes the lung to environmental antigens, microorganisms, gases and microparticles, including pollutants. Air pollution is a global concern. Nearly the entire world’s population breathes air that contains higher levels of pollutants than the World Health Organization considers safe, with low- and middle-income countries experiencing the highest exposure levels [2]. Air pollution causes one in every six deaths globally and was responsible for an estimated 9 million deaths in 2019 alone [3], while pollution-associated death rates have risen by over 66% since 2000 [3]. This adverse health effect is greater than that of terrorism, war, infectious diseases, drugs, and alcohol, and on par with smoking [3].

A major air pollutant is the reactive oxygen molecule ozone. While stratospheric ozone has a protective function, as it absorbs ultraviolet light [4], ground-level ozone is detrimental to human health and is estimated to account for 489,000 deaths globally every year [5]. This number is expected to rise because of increased ground ozone levels due to increasing ambient temperatures [6]. Ozone exposure induces and exacerbates lung injury and is associated with increased morbidity and mortality from lung diseases [7]. For example, chronic obstructive pulmonary disease (COPD)-related deaths attributable to ozone exposure have increased by almost 25% over the past decade [5]. It is therefore important to understand the mechanism of ozone-induced injury.

The airway epithelium is the first tissue that gets into contact with and is injured by inhaled ozone. Airway epithelial cells create a physical barrier through tight junctions, clear inhaled particles and microbes by mucociliary transport, secrete antimicrobial peptides, cytokines, and chemokines that coordinate innate and adaptive immune responses and contribute to tissue repair, and regeneration after injury. Epithelial cell injury impairs mucosal barrier integrity, host defense, and repair [8,9]. Disruption of epithelial integrity is a hallmark of various chronic lung diseases, including asthma, COPD and pulmonary fibrosis [10]. Ozone exposure leads to oxidation and breakdown of airway cellular and matrix molecules due to its oxygen radical properties [11]. In the extracellular matrix, ozone-induced lung injury has been associated with the breakdown of the glycosaminoglycan, hyaluronan (HA) [12,13]. HA is the most abundant extracellular matrix glycosaminoglycan in the human body and is an important component of the glycocalyx in many epithelial surfaces, such as the skin [14] and the luminal surface of the conducting airways [15]. HA is involved in both physiological (e.g., tissue hydration [16]) and pathological (e.g., inflammation [17]) pathways. In healthy tissue it is present as high molecular weight HA (HMW HA, molecular weight > 1000 kDa), which is thought to have anti-inflammatory effects [18]. However, during injury, there is a generation of shorter HA fragments (sHA, generally <250 kDa) which are proinflammatory [18,19,20]. HA effects on airway epithelia are mediated by receptors such as CD44 and RHAMM [21,22]. CD44 is a widely expressed transmembrane HA receptor that mediates cell adhesion, migration, and signaling. RHAMM functions at the cell surface as well as intracellularly, and regulates immune activation, cytoskeletal organization, cell motility, proliferation, and mitotic spindle dynamics. We have previously shown that sHA mediates airway inflammation via CD44 while HMWHA ameliorates ozone-induced airway inflammation and hyperresponsiveness [23]. We have also previously demonstrated that HA is required for ozone-induced alveolar epithelial cell proliferation [24]. However, the role of HA in ozone-induced lung airway epithelial injury has not been elucidated.

In this study, we utilized murine and human airway epithelial cultures differentiated at air–liquid interface (ALI) to assess cell integrity and transcriptomic responses following ozone exposure and HMWHA treatment. Our findings demonstrate that ozone induces airway epithelial injury that shares important transcriptomic signatures between murine and human airway epithelia. Interestingly, the HA receptors CD44 and RHAMM mediate opposing effects on murine epithelial response to ozone-induced injury, while exogenous administration of HMWHA effectively mitigates epithelial injury via activation of the innate immune receptor TLR5, a receptor with important functions in mucosal defense, epithelial barrier integrity and antimicrobial activity [25,26,27]. This suggests an important role for HA in the generation of ozone-induced epithelial injury and suggests potential HA-mediated therapies to limit these effects.

## 2. Materials and Methods

### 2.1. Experimental Animal Information

C57Bl/6J mice (Strain #000664), *Cd44*-deficient mice (Strain #005085), *Tlr4*-deficient mice (Strain #029015) and *Tlr5*-deficient mice (Strain #028909) were purchased from the Jackson Laboratory (Bar Harbor, ME, USA). *Rhamm*-deficient mice were graciously donated by Rashmin Savani and Eva Turley [28]. All transgenic mice were whole-body knockouts. Mice were maintained at the NIEHS Vivarium under a 12 h light–dark cycle and were provided chow and water ad libitum until euthanized for cell harvest as described below.

### 2.2. Establishment of Murine Differentiated Air–Liquid Interface (ALI) Airway Cell Culture

All experiments were approved by the NIEHS animal use and care committee. Mouse tracheas were obtained from male C57Bl/6J, *Tlr4*-, *Tlr5*-, *Rhamm*- and *Cd44*-deficient mice (*n* = 13 per group) aged between 12 and 14 weeks. The mice were euthanized by CO_2_ inhalation in accordance with institutional animal care guidelines, and tracheas were carefully excised and stored in a sterile Ham’s F12/penicillin-streptomycin (PS)/folic acid (FA) medium on ice. Tracheal epithelial cells were isolated as previously published [29]. Briefly, tracheas were cleaned of surrounding tissue under aseptic conditions and then incubated at 4 °C overnight in 0.15% Pronase mixed in Ham’s F12/PS/FA. The tracheas were rinsed in a solution of 20%FBS Ham’s F12/PS/FA. The Pronase solution was combined with the rinse solution and spun down (390 g, 5 min, 4 °C). The pellet was resuspended in DNAse (0.5 mg/mL) (Sigma-Aldrich, St Louis, MO, USA), incubated for 5 min on ice, centrifuged (390 g, 5 min, 4 °C), resuspended in MTEC Plus media and incubated for 3 h on a Primaria dish for negative selection of fibroblasts. The suspension was spun down again (390 g, 5 min, 4 °C), resuspended in MTEC Plus media (Sigma-Aldrich, St Louis, MO, USA), and the cells were counted using a Bio Rad TC20 automated cell counter (Bio Rad, Hercules, CA, USA). The cells were subsequently plated on collagen type I pre-coated plates at a seeding density of 10^5^/transwell (12 mm insert) and incubated at 37 °C in a humidified atmosphere of 5% CO_2_ until confluence was reached, typically within 3–5 days. Then, the apical medium was removed creating the air–liquid interface. The cells were maintained at the air–liquid interface for 10–14 days to allow them to differentiate, and basal media were exchanged every two days. During the differentiation process cells were washed with phosphate-buffered saline (PBS) every two days when the medium was changed to remove accumulated mucus. Cells were monitored several times a week macroscopically and microscopically until used.

### 2.3. Establishment of Human Differentiated Airway Cell Culture

Human primary bronchial epithelial cells were purchased from Lonza. The cells were grown in Basal Epithelial growth media (Lonza, Walkersville, MD, USA) in T175 until they reached 70% confluence under standard cell culture conditions at 37 °C in a humidified atmosphere with 5% CO_2_. Cells were seeded at a density of 1.5 × 10^5^/well on permeable cell culture inserts coated with either rat tail collagen I or bovine collagen I (PureCol, Advanced Biomatrix, Carlsbad, CA, USA) in submerged conditions with bronchial epithelial growth media (Supplemental Methods) until confluence was reached, typically within 3–5 days. Then, cells were treated as described above. The cells were maintained at the air–liquid interface for 21–28 days to allow them to differentiate prior to exposure. For some experiments, commercially available human ALI cultures were obtained from the biotechnology company MatTek Life Sciences (Ashland, MA, USA) and utilized according to the manufacturer’s instructions with manufacturer supplied reagents and media.

### 2.4. Cell Treatment

Fully differentiated murine and human cells were treated with PBS or HMW HA solution (3 mg/mL, IBSA, CH-6926 Collina d’Oro-Montagnola, Switzerland) for 2 h. Subsequently the solution was removed, and the cells were exposed to free air or ozone (400 ppb, MedTec, Durham, NC, USA [30]) for 1 h (murine culture) or 2 h (human culture). After this timepoint (i.e., 1 h for the murine cultures and 2 h for the human cultures), barrier function was evaluated vie TEER or FITC–dextran measurements, and cells and apical washings were harvested for RNA sequencing, HA abundance, and HA size analyses. For some experiments involving Tlr5 activation, we used a flagellin analog pure Tlr5 agonist, graciously donated by Andrei Gudkov, Roswell Park Comprehensive Cancer Center, at a dose of 0.5 μg/mL [31].

### 2.5. Analysis of Epithelial Barrier Permeability with Transepithelial Electrical Resistance (TEER)

TEER across the epithelial monolayer was measured directly after ozone exposure by using the Millicell electrical resistance system ERS-2 (Merck Millipore, Burlington, MA, USA) per manufacturer’s instructions. After ensuring that the ALI culture layers were confluent we equilibrated the plate to room temperature. After sterilizing the electrode, we place the shorter prong into the apical chamber and the longer prong into the basolateral chamber, holding the probe perpendicular to the membrane. We recorded the resistance (Ohm) after the reading stabilized, ensuring the electrode did not touch the membrane itself. TEER values were calculated as TEER = (R_sample_ − R_blank_) × A_membrane_ whereby R_blank_ is a cell-free insert, and expressed in Ω × cm^2^.

### 2.6. Analysis of Epithelial Barrier Permeability with FITC–Dextran

4 kDa FITC–dextran (Sigma Catalog number 46944, Sigma Aldrich, USA) was diluted to a final concentration of 2 mg/mL in PBS. 200 μL of FITC–dextran were added to the apical side of air–liquid interface cultures after O_3_ exposure and incubated at 37 °C, 5% CO_2_ for up to 3 h. Media were collected from the basolateral side of the air–liquid interface culture and fluorescence was evaluated under excitation wavelength 492 nm, emission wavelength 520 nm, using a Varioskan Lux Plate Reader (Thermo Fisher, Waltham, MA, USA).

RNA isolation and sequencing, and reverse transcription polymerase chain reaction (RT-PCR) Total RNA was extracted from human and mouse tissue samples 3 h post ozone exposure using the Qiagen RNeasy mini kit (Qiagen, Germantown, MD, USA) according to the manufacturer’s protocol. The concentration of the RNA was assessed using Qubit 4 fluorometer (Thermo Fisher, Waltham, MA, USA). RNA integrity was evaluated using the Agilent 4200 TapeStation System to ensure an RNA integrity number (RIN) greater than 8 RNAseq libraries were generated by the NIEHS Epigenomics Sequencing Core using Illumina Stranded mRNA kit (Illumina, San Diego, CA, USA). The libraries were validated using the TapeStation 4200 and the Qubit 4 fluorometer and submitted for sequencing. Isolated RNA from murine and human samples was transcribed to complementary DNA using the High-Capacity cDNA reverse transcriptase kit. cDNA was then amplified using the Power SYBR Green PCR master mix (Applied Biosystems, Carlsbad, CA, USA). Primer sequences for the specific cytokines or chemokines tested are shown in Supplemental Methods.

### 2.7. Bioinformatic Analyses

Raw sequencing data were trimmed to remove adaptors and poor-quality reads (those with an average quality score < 20) using Trim-galore (version 0.6.10). The cleaned reads were then aligned to the mouse mm39 reference genome or the human hg38 reference genome using the STAR splice-aware aligner (version 2.6.0 c) [32] with default parameters. Ambiguous reads that mapped to multiple regions in the genome and reads with a MAPQ score less than 10 were removed. Gene quantification was performed using Subread featureCounts (version 2.0.6) [33] with the mm39 RefSeq gene annotation. Genes without read counts in all samples were excluded from further analysis. The remaining genes were analyzed using the edgeR package (version 4.0.16) [34] to identify differential expression between controls and treated samples. Benjamini and Hochberg’s method was applied to control the false discovery rate. Genes were considered significantly differentially expressed if they met the following criteria: detected in at least one sample (RPKM > 1), fold change exceeding 2, and an adjusted *p*-value < 0.05.

Pathway analysis was conducted by uploading significant differentially expressed gene lists to Ingenuity Pathway Analysis (IPA, Ingenuity, Redwood City, CA, USA) for comprehensive pathway exploration. The unfiltered expression dataset was subjected to Gene Set Enrichment Analysis (GSEA, version 4.3.2) [35] using the Molecular Signatures Databases MSigDB (version 2024.1.Mm, or version 2024.1.Hs for mouse and human sequences respectively). Enrichment significance was evaluated through 1000 permutations against hallmark gene sets. Gene sets with an FDR q-value < 0.05 were considered statistically significant.

### 2.8. Enzyme-Linked Immunosorbent Essay (ELISA)

HA levels in murine cell culture apical wash were measured by DuoSet ELISA (R&D Systems, Minneapolis, MN, USA) per manufacturer’s instructions, using a Multiscan EX microplate spectrophotometer (ThermoFisher Scientific). Linear regression analysis was performed to interpolate values from the standard curve.

### 2.9. ZO1 Staining

The apical surface of ALI cultures was washed twice with 1XPBS to remove excess mucus, then cultures were fixed for 30 min in 4% paraformaldehyde (PFA) before changing to 1XPBS. Fixed cultures were first permeabilized with 0.2% TBST (Tris Buffered Saline supplemented with Triton-X-100) for 30 min at room temperature, washed twice with 1XTBST, then non-specific proteins blocked for 1 h in blocking diluent (1% BSA, 10% normal donkey serum in 1XTBST). Primary antibody (mouse anti-ZO-1, Thermo Fisher Scientifc, Durham, NC, USA) was added at a dilution of 1:100 in blocking diluent and incubated at 4 °C for 48 h. After washing in 1XTBST twice, secondary antibody (1:100; donkey anti-mouse IgG 594) was added along with DAPI (1:1000) and incubated for 1 h at room temperature, then washed twice in 1XPBS. Cover slips were affixed with Prolong Diamond Mount and 20× images collected with a Zeiss LSM 880 inverted confocal microscope and 10× tile scans with a Zeiss LSM 780 inverted confocal microscope (Carl Zeiss, Inc, Oberkochen, Germany) with Zen software (version 3.13). The images were brought into FIJI (1.54f), Labkit [36] was used to identify the ZO1 matrix, and the segmentation result was applied to the raw data to obtain the area and mean intensity of the ZO1. Imaris 9.9 (Oxford Instruments plc, Abington, UK) was used to help visualize the two layers of cells.

### 2.10. HA Size Determination via Solid-State Nanopore Analysis

Affinity extraction [37] and nanopore analysis [38,39] of HA were performed following previously established protocols with minor adaptations. Briefly, samples were incubated at room temperature for 60 min with 1.5 mg of magnetic beads functionalized with biotinylated Versican G1 domain (bVG1; Cat. G-HA02, Echelon Biosciences, Salt Lake City, UT, USA) immobilized on streptavidin-coated Dynabeads™ M-280 (Cat. 11205D, Thermo Fisher, Waltham, MA, USA). Following incubation, beads were magnetically separated and washed to eliminate unbound components. The captured HA was subsequently released by incubating the beads for 60 min at room temperature in 40 µL of measurement buffer consisting of 6 M LiCl, 10 mM Tris, and 1 mM EDTA (pH 8.0). Recovered HA samples were analyzed directly with commercially fabricated solid-state nanopore sensors (P/N: NXPR4001Y-10 nm-ABX1, Norcada, Inc., Edmonton, AB, Canada). Translocation measurements were performed under a 300 mV bias while transmembrane current was recorded at a rate of 200 kHz using a 100 kHz four-pole Bessel filter. Data were collected and analyzed with custom LabVIEW programs (National Instruments, Austin, TX, USA) with which an additional 5 kHz low-pass filter was applied during analysis. All nanopore diameters were between 7.5 and 8.5 nm, calculated from the measured current-voltage curve and assuming [40] an effective pore thickness equal to 1/3 the full membrane thickness (20 nm). Translocation signals (events) were identified by applying a threshold corresponding to 5 times the standard deviation of the baseline root-mean-square (RMS) noise and a minimum duration of 25 μs. Measurement of a calibration on each device prior to sample analysis enabled the conversion of translocation event areas to molecular weights (MWs) on a molecule-by-molecule basis [38]. The calibration standard was a mixture of three quasi-monodisperse HAs (111, 545, and 1071 kDa; Hyalose, LLC, Oklahoma City, OK, USA) synthesized chemoenzymatically as described elsewhere [41]. Translocation events corresponding to HAs between 50 kDa and 10 MDa were used to construct MW distributions.

### 2.11. Tlr5 Activity Assay

*HEK-Blue™ mTLR5* and *HEK-Blue™ Null1-v cells* (Invivogen, Carlsbad, CA, USA) were cultured as per manufacturer’s specifications in RPMI DMEM, 4.5 g/L glucose, 2 mM L-glutamine, 10% (*v/v*) fetal bovine serum (FBS), Pen-Strep (100 U/mL-100 μg/mL), 100 µg/mL Normocin™ or with heat inactivated FBS for Null cells. The cells are maintained and cultured under selection with 30 µg/mL of blasticidin and 100 µg/mL of Zeocin^®^ for Hek-Blue mTLR5 cells or 100 µg/mL of Zeocin^®^ for Hek-blue Null cells. Cells were assayed by adding 20 µL of each test solution to well of a flat-bottom 96-well plate. Absorption of null cells was subtracted from the TLR5 expresing cells, and the result was reported as an indicator of TLR5 activation.

### 2.12. Statistical Analysis

GraphPad Prism 10 (Dotmatics, Boston, MA, USA) was used for statistical analysis. Student’s *t*-test or ANOVA with post hoc correction for multiple comparisons was utilized as indicated. *p* values < 0.05 were considered statistically significant.

## 3. Results

### 3.1. Ozone Exposure Impairs Lung Epithelial Cell Integrity

**Figure 1:**
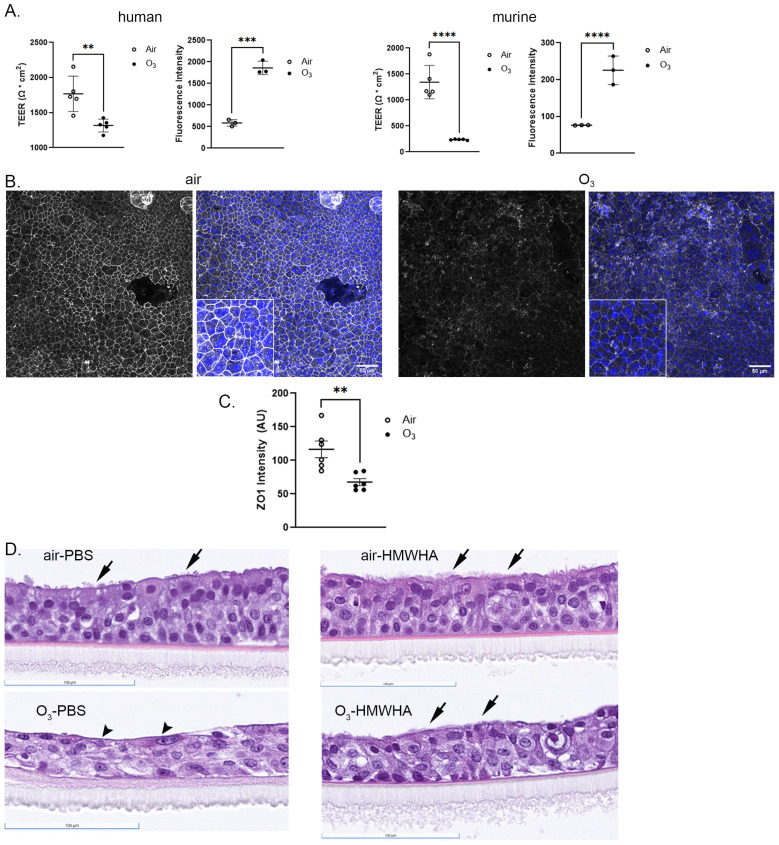
Ozone exposure leads to epithelial injury and loss of integrity. (**A**) Transepithelial electrical resistance (TEER, left) and FITC–dextran permeability (right) measurements of fully differentiated murine and human ALI cultures immediately after in vitro exposure to air or ozone (400 parts per billion) for 1 (mouse) or 2 (human) hours. (**B**) ZO1 staining post air and ozone exposure. Inserts: higher magnification of epithelial layer. (**C**) ZO1 intensity quantification in murine ALI culture. ZO1 = Zona occludens 1, AU = arbitrary units. (**D**) Hematoxylin–Eosin staining of ALI cultures after exposures and treatments as labeled. Experiments repeated at least twice. ** *p* < 0.01, *** *p* < 0.001, **** *p* < 0.0001, Student’s *t*-test.

We first evaluated the comparative effect of ozone on epithelial integrity using trans-epithelial electrical resistance (TEER), a commonly used quantitative technique that assays the integrity of cell–cell connections in in vitro models of epithelial/endothelial mono- and multilayers [42]. We used human ALI cultures as they are the most appropriate cell system to evaluate effects of our exposures in human biology, and murine ALI cultures, as the most common model system used in airway biology, to establish the degree to which murine and human biology converges in this exposure system. TEER measurements in murine ALI culture showed that a 1 h ozone exposure caused a significant reduction in TEER to below 50% of initial values, in contrast to minimal changes in the air-exposed group (Figure 1A). Ozone exposure for 2 h also caused a significant TEER decrease in human ALI culture (Figure 1A). To confirm our TEER findings, we evaluated epithelial barrier function using a second method, i.e., diffusion of FITC–dextran from the apical surface to the basal chamber of the ALI culture system. The results were identical to TEER results, demonstrating a significant reduction in epithelial integrity (increase in FITC–dextran fluorescent intensity detected in the basal media) after ozone exposure. To further investigate the integrity of tight junctions in murine ALI culture, immunocytochemistry images of the Zona occludens 1 (ZO1) protein were taken, along with 4′,6-Diamidin-2-phenylindol (DAPI) counterstaining to visualize cell nuclei (Figure 1B,C). There was significant reduction in ZO1 staining intensity after ozone exposure in the murine model (Figure 1B,C). In histology, ozone exposure resulted in flattening of the epithelial layer and loss of ciliated cells (Figure 1D, arrowhead), in contrast to air-exposed or hyaluronan-treated cells (Figure 1D, arrows). These data in aggregate support that ozone induces epithelial injury in murine and human ALI cultures.

### 3.2. Ozone Exposure Induces Comparable Transcriptomic Changes in Murine and Human ALI Culture

**Figure 2:**
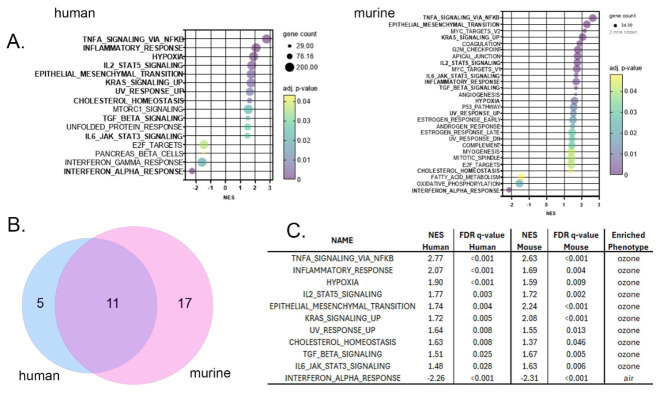
Transcriptomic effects of ozone exposure in human and murine differentiated airway epithelia. (**A**) Gene Set Enrichment Analysis (GSEA) of human and mouse differentiated airway epithelial cell cultures differentiated at an air–liquid interface 3 h post in vitro ozone exposure. (**B**) Venn diagram of overlapping up- and downregulated gene expression pathways in GSEA of human and murine airway epithelial after ozone exposure. (**C**) List of common pathways between human and murine epithelia. NES = normalized enrichment score. FDR = false discovery rate. Enriched phenotype: condition (air or ozone exposure) in which this pathway is relatively upregulated.

To further elucidate the transcriptional response of fully differentiated airway epithelial cells to ozone, we performed RNA sequencing of cells harvested 3 h after the completion of ozone exposure. We found that ozone exposure resulted in 7780 differentially expressed genes (DEGs) in mouse and 2262 DEGs in human ALI cultures. Gene Set Enrichment Analysis (GSEA) of the transcriptomic gene set showed that ozone exposure was associated with a strong inflammatory response, epithelial damage, oxidative stress and DNA damage responses, metabolic impairment and a possible transition to a more mesenchymal-like state (Figure 2A). Notably, there was significant overlap between human and mouse in the airway epithelial response to ozone. Eleven of 16 (69%) significantly affected human gene sets and 11 of 28 (39%) murine gene sets were expressed in common between the two species using an FDR q-value cutoff < 0.05 (Figure 2B). Common pathways that were identified in human and murine airway cells (Figure 2C) included inflammatory responses: for example, TNFα signaling via NFκB was upregulated with a normalized enrichment score (NES) > 2.5 in both human and murine ALI culture, IL2/STAT5 signaling was upregulated with a NES > 1.5 in both species, and IL6/JAK-STAT3 signaling was similarly upregulated. Another common process centered around remodeling: the epithelial–mesenchymal transition and TGFβ1 gene sets were upregulated to a similar degree. Pathways related to oxidative stress and DNA damage (Hypoxia, UV response, KRAS signaling) were also induced in both species. Lastly, interferon-associated pathways were downregulated after ozone exposure in both species. In summary, our RNA-sequencing analysis demonstrates that ozone elicits strong injury responses in airway epithelial cells that are comparable in murine and human models, indicating conserved mechanisms. This supports that murine ALI culture can reliably model human epithelial injury mechanisms and processes after ozone exposure.

### 3.3. Differential Effects on Ozone-Induced Airway Epithelial Inflammation and Integrity by HA Receptors Cd44 and Rhamm

**Figure 3:**
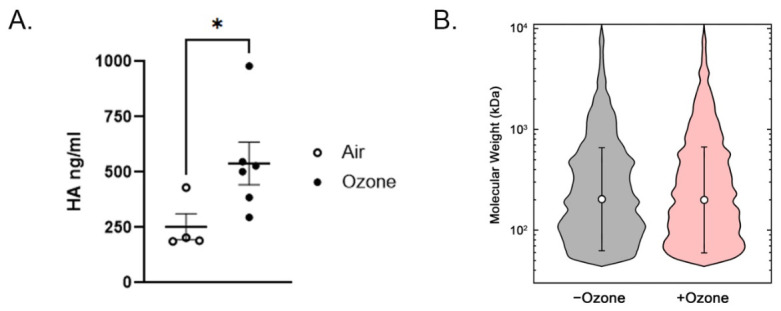
(**A**) Induction of epithelial HA expression 3 h after ozone exposure. (**B**) Size analysis of HA from apical washes of airway epithelial cell cultures before and 3 h after ozone exposure. * *p* < 0.05, Student’s *t*-test.

To further investigate ozone-mediated airway epithelial injury, we generated airway epithelial ALI cultures from tracheas of genetically modified mice. We have previously shown that fragmentation of HA mediates ozone-induced lung inflammation and airway hyperresponsiveness in vivo [23]. We first evaluated the HA size in epithelial lining fluid from ALI cultures before and after ozone exposure. We found that ozone exposure to ALI cultures increases HA expression (Figure 3A). The majority of HA (by molecule count) was in the lower molecular weight, with a peak molecule frequency approx. at 100 kDa. HA size was not significantly changed by ozone exposure (Figure 3B). We then investigated the expression of HA receptors. HA receptors *CD44*, *LAYN* and *HMMR* were expressed in differentiated human airway epithelial cultures (Table 1). Of these, murine airway epithelial *Cd44* and *Hmmr* expression were significantly changed after ozone exposure in murine epithelial cells. We therefore evaluated the role of CD44 and RHAMM in ozone exposure using genetically deficient murine tracheal epithelial cell cultures.

**Figure 4:**
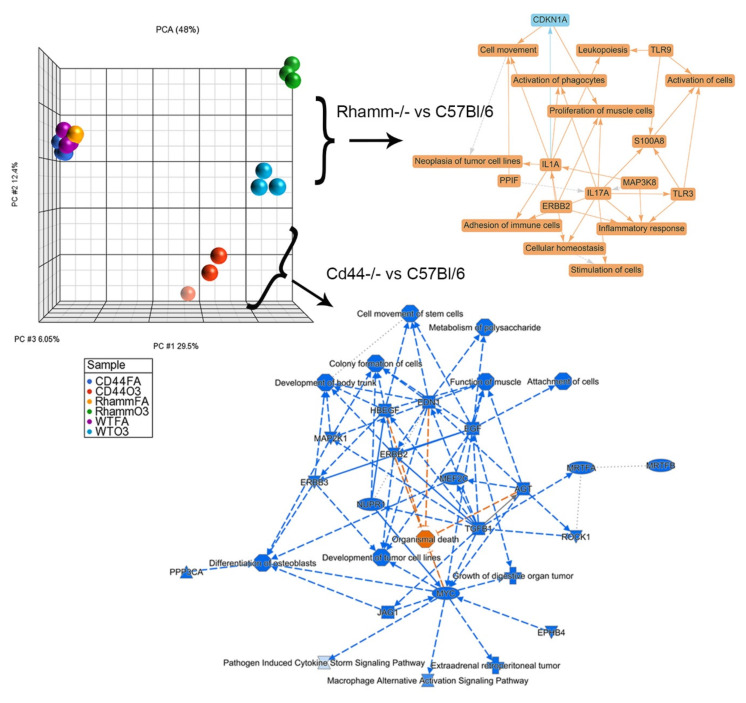
Transcriptomic effects of ozone exposure dependent on Cd44 and Rhamm signaling. Principal component analysis (PCA) of C57BL/6J (wildtype, WT) cells, *Cd44*-deficient cells (CD44 KO) and *Hmmr*-deficient cells (RHAMM KO) before and after in vitro ozone exposure with associated network analysis of CD44KO cells vs. WT cells and RHAMM KO cells vs. WT cells. Blue color in the network analysis denotes downregulated pathways, while orange color denotes upregulated pathways.

Absence of CD44 or RHAMM did not affect the decrease in TEER observed after ozone exposure (Supplementary Figure S1). However, when we evaluated the transcriptomic response to ozone, principal component analysis (PCA) suggested that absence of CD44 or RHAMM led to significantly divergent responses (Figure 4). We found that CD44 deficiency generally led to a downregulation of transcriptional responses, mostly centered around nodes Myelocytomatosis (MYC), transforming growth factor β1 (TGFβ1) and growth factor receptors erythroblastosis oncogene B (ERBB)2 and ERBB3 (Figure 4). By contrast, RHAMM deficiency led to an upregulation of transcriptional pathways centered around responses in the IL1A and IL17A pathway while ERBB2 was upregulated in direct contrast to the CD44 transcriptomic response (Figure 4). These results support that HA activation of CD44 may lead to cell activation and inflammation, while activation of RHAMM may lead to homeostatic responses, suppressing inflammation.

We evaluated the differentially expressed genes (DEGs) for the RHAMMKO vs. wildtype and CD44KO vs. wildtype comparisons. RHAMM deficiency amplified the ozone-driven injury response. The strongest upregulated genes include Myc, Ereg, Snai2, Cxcl10, Cxcl1, Cxcl2, Icam1, Il11, Tnf, Trp63, and Jag1, suggesting an amplified injury and inflammation response. The strongest downregulated genes included Wnt7b, Ezr, Foxj1, Tppp3, and Krt8, suggesting a relative loss of mature epithelial features. In aggregate, these data suggested that RHAMM deficiency exaggerates epithelial inflammatory signaling and epithelial remodeling after ozone exposure. CD44 deficiency suggested a different transcriptomic pattern with blunting of the canonical WT ozone response program, especially genes linked to Notch, EGFR and epithelial repair, such as Jag1, Errfi1, Jun, Dusp5, Dusp10, Edn1, Hbegf, Trp63, Krt17, Sox9, Snai2, Myc, Spry2, which were downregulated in CD44 KO compared to WT.

**Figure 5:**
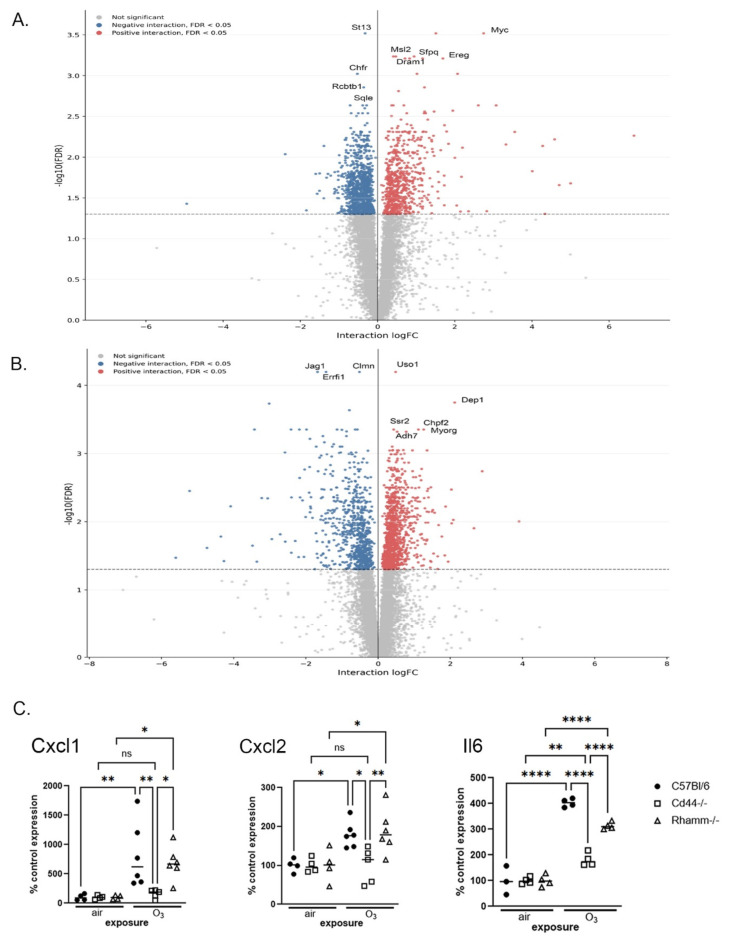
Differentially expressed genes (DEG) of RHAMM- and CD44-deficient cells compared to C57Bl6 cells. (**A**) DEG Volcano plot for RHAMM-deficient cells compared to C57Bl6. (**B**) DEG Volcano plot for CD44-deficient cells compared to C57Bl6. (**C**) Expression of inflammatory chemokines and cytokines before and after ozone exposure in C57Bl/6, CD44 deficient, and RHAMM-deficient murine epithelial cell cultures. * *p* < 0.05, ** *p* < 0.01, **** *p* < 0.0001, ns = nonsignificant, ANOVA with multiple comparisons correction. Dashed horizontal line = significance cutoff.

To validate these findings, we evaluated the expression of *Cxcl1*, *Cxcl2* and *Il6* by RTPCR. We selected these as representative of the inflammatory response that were also found to be top DEGs in RNA-sequencing analysis mentioned above. We found that CD44-deficient cells had blunted induction of these cytokines, while RHAMM-deficient cells had comparable or somewhat higher responses to wildtype cells (Figure 5C). In aggregate, these results suggest that HA signaling effects in ozone-induced epithelial injury may differ when mediated by different receptors.

### 3.4. High Molecular Weight HA Ameliorates Lung Epithelial Cell Integrity After In Vitro Ozone Exposure via TLR4 and TLR5 and Independent of CD44 and RHAMM

**Figure 6:**
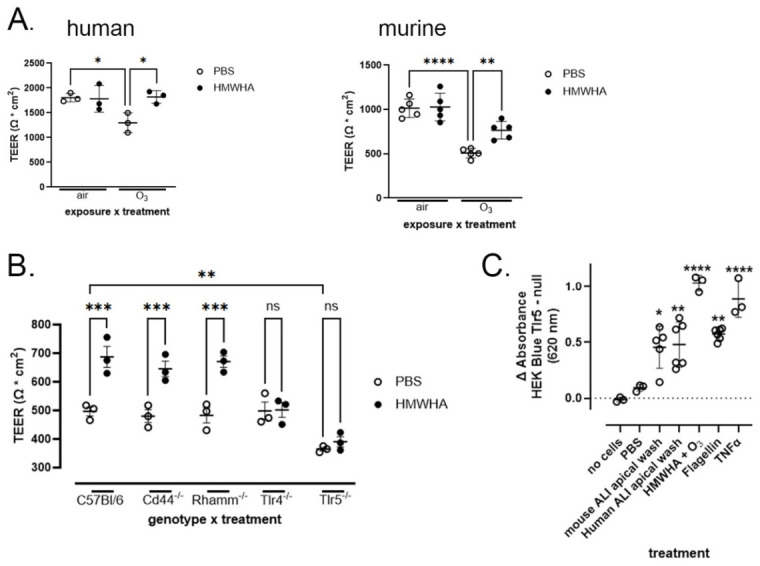
HMWHA promotes epithelial integrity after ozone exposure through innate immune receptors TLR4 and TLR5. (**A**) HMWHA ameliorates reduction in TEER after in vitro ozone exposure (400 ppb) in ALI differentiated human and murine airway epithelial cell cultures. (**B**) HMWHA effect on ozone-induced TEER is preserved in CD44- and RHAMM-deficient airway epithelial cells but not in TLR4- or TLR5-deficientg cells. Note that TLR5-deficient cells have exacerbated loss of airway epithelial integrity as assayed by TEER in contrast to TLR4-deficient cells. (**C**) TLR5 is activated by HA and apical washes of human and murine ALI cultures. Flagellin and TNFα are positive controls. Experiments repeated at least twice. * *p* < 0.05, ** *p* < 0.01, *** *p* < 0.001, **** *p* < 0.0001, ANOVA with multiple comparisons correction. For Panel (**C**) asterisks refer to comparisons with the PBS control group. C57Bl/6 = “wildtype” background strain.

We have previously shown that high molecular weight HA ameliorates ozone-induced lung injury and airway hyperresponsiveness in vivo [23]. We now evaluated the effect of HMWHA in ozone-induced airway epithelial injury in vitro. We found that HMW HA treatment significantly ameliorates the loss in airway epithelial cell integrity after ozone exposure in human as well as murine airway epithelial cell cultures (Figure 6A). In the air-exposed group there was no effect of HMWHA.

We then evaluated whether the beneficial HMWHA effect was mediated by specific receptor signaling. Because we have found that TLR5 mediates HA signaling in complex with TLR4 [43] and protects airway epithelia from injury [31], we evaluated TLR4 and TLR5 alongside CD44 and RHAMM. Interestingly, we found that CD44 and RHAMM did not mediate HMWHA protection from loss in epithelial integrity after ozone exposure, as wildtype, CD44- and RHAMM-deficient cells exhibited similar increase in TEER with HMWHA treatment after in vitro ozone exposure (Figure 6B). However, TLR4- and TLR5-deficient failed to respond to HMWHA treatment. Notably, TLR5-deficient cells had a significantly greater decline in TEER after in vitro ozone exposure compared to wwildtypeand TLR4-deficient cells which remained low even after HMWHA treatment (Figure 6B). HA signaling via TLR4 is well-established [44,45]. To further confirm that TLR5 is indeed activated by HA in our system, we utilized HEK-Blue™ TLR5 reporter cells. We found that HA and apical washes from both murine and human ALI cultures induced TLR5 activation (Figure 6C). In aggregate, these results suggested that HMWHA ameliorates loss in epithelial integrity via TLR4 and TLR5.

### 3.5. High Molecular Weight HA Ameliorates Ozone-Induced Inflammation and Epithelial Injury

**Figure 7:**
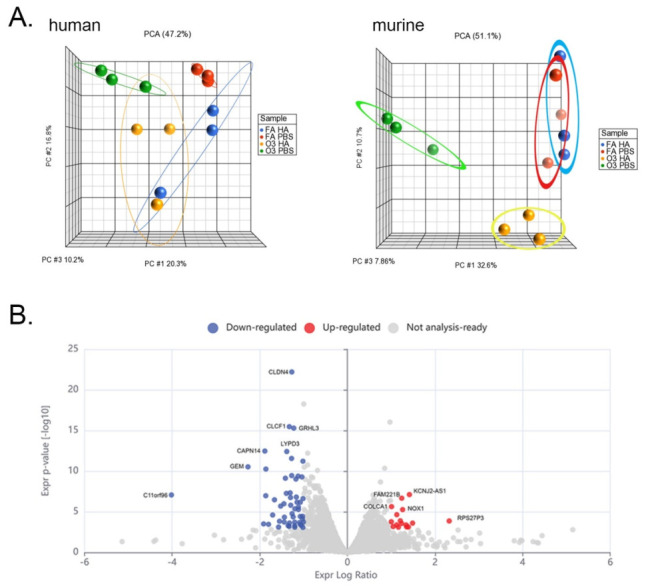
Effect of HMWHA on human and murine airway epithelial gene expression after ozone exposure. (**A**) Principal component analysis (PCA) of human and murine gene sets shows a clear shift in gene expression with HMWHA treatment on in vitro ozone-exposed airway epithelial cells. (**B**) Volcano plot of human ozone-exposed, HMWHA-treated vs. vehicle (PBS)-treated airway epithelial cells.

We performed RNA sequencing of human airway epithelial after in vitro ozone exposure with or without HMWHA treatment, to evaluate the effect of HMWHA on the transcriptional response to ozone exposure. Principal component analysis revealed that HMWHA largely reversed the transcriptomic response to ozone exposure in mouse and human airway epithelial cells, with ozone-HA-treated cells clustering very close to the air-exposed cells (Figure 7A). Furthermore, HMWHA significantly suppressed expression of proinflammatory and injury responses in both human and murine airway epithelial cell cultures (Figure 7B). Analysis of DEGs suggested that HMWHA strongly ameliorates the ozone response of epithelia. For example, among genes significant in both ozone vs. air and HMWHA-ozone vs. PBS-ozone, 258 of 262 DEGs were changed in the opposite direction. The genes suppressed by HMWHA are enriched for epithelial barrier (e.g., CLDN4), polarity (LYPD3, GHRL3, CLCF1), and ER-stress functions (HERPUD1), suggesting that HMWHA acts upstream of key epithelial injury responses. The biggest HMWHA-induced genes by effect size are POSTN, COL3A1, and COL6A3, supporting a specific remodeling signature.

**Figure 8:**
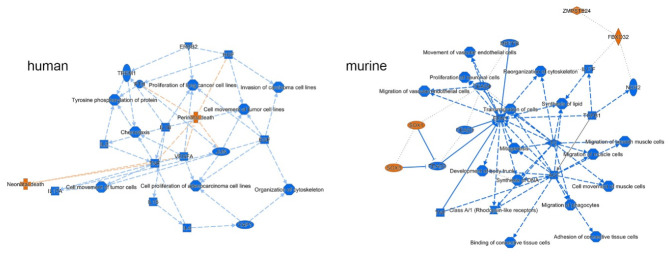
Effect of HMWHA on human and murine airway epithelial gene expression networks after ozone exposure. Network analysis of human and murine airway epithelia, HMWHA-treated vs. vehicle (PBS)-treated, shows a downregulation (blue) of most pathways. Orange = upregulation.

Network analysis of the pathways affected by HMWHA treatment, shows that both in human and murine airway epitheli there is a strong downregulation of injury response pathways, e.g., proliferation, chemotaxis and migration pathways which are suppressed by HMWHA in both murine and human cells (Figure 8).

**Figure 9:**
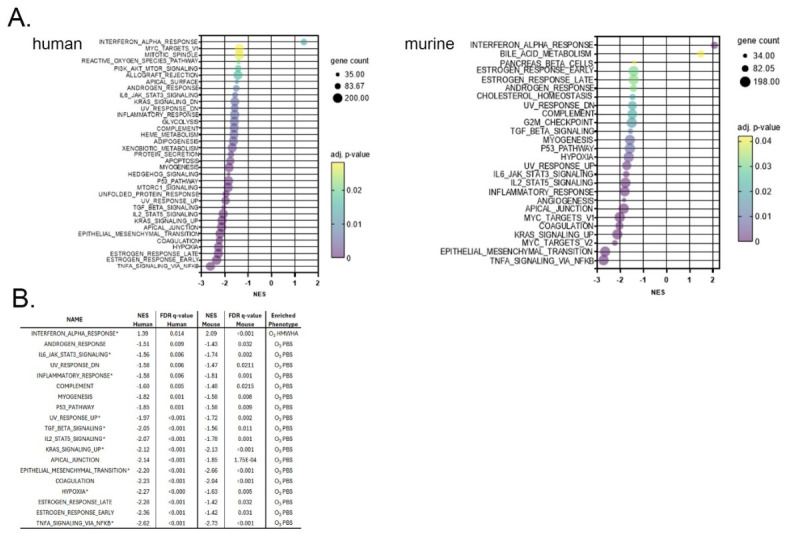
(**A**) GSEA identifies differentially regulated pathways in human and murine airway epithelial cells. (**B**) Common pathways that are differnetially regulated by HMWHA in both human and murine airway epithelial cells. * Common gene sets between human and mouse, that are changed by HMWHA in the opposite direction from ozone exposure (e.g., downregulated by HMWHA in ozone exposure, but upregulated with ozone exposure alone, compare Figure 2C).

**Figure 10:**
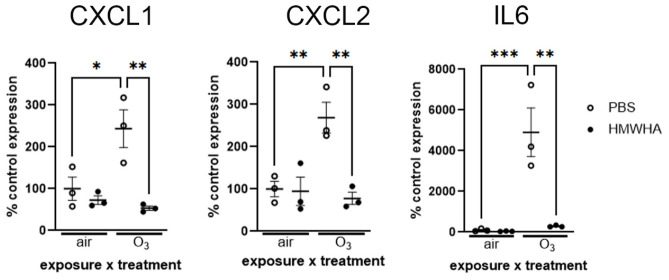
Mouse Cxcl1, Cxcl2 and Il6 RTPCR after in vitro air or ozone exposure to airway epithelial cells with and without HMWHA administration. * *p* < 0.05, ** *p* < 0.01, *** *p* < 0.001, ANOVA with multiple comparisons correction.

GSEA confirmed and expanded the principal component and network analysis results. Murine airway epithelial dataset analysis showed that HMWHA led to a strong downregulation in the inflammatory response and airway remodeling process after ozone exposure (Figure 9A). Additionally, lower oxidative stress and DNA damage levels showed a clear contrast to the ozone-exposed, PBS-treated group. Human dataset analysis replicated the murine results with a clear decrease in the inflammatory, airway remodeling and oxidative stress and cellular damage response to ozone after HMWHA treatment (Figure 9A). We once again identified common pathways between human and murine airway epithelial cells that were differentially regulated by HMWHA (Figure 9B). We confirmed the significant effect of HMWHA on ozone-induced inflammation by RTPCR for Interleukin 6, C-X-C motif chemokine ligand 1 (Cxcl1) and Cxcl2, which we had earlier identified as significantly induced by in vitro ozone exposure (compare Figure 5C). We found that HMWHA significantly downregulated the ozone-induced increase for these cytokines (Figure 10). To confirm that HMWHA acts via Tlr5 signaling, we compared the transcriptomic response of human ALI cells to HMWHA and a Tlr5 agonist. Ingenuity Pathway Analysis suggested that there were very few DEGs for this comparison (Supplementary Figure S2). In aggregate, these data support that HMWHA protects against ozone-induced airway epithelial inflammation and improves general cell conditions in ozone-induced injury in airway epithelial cells.

## 4. Discussion

In this study we investigated the role of HA signaling in ozone-induced injury in murine and human airway epithelial cell cultures differentiated at air liquid interface. We found that ozone impairs airway epithelial cell integrity and induces an inflammatory response which is ameliorated by HMWHA. Additionally, transcriptomic data suggest HA receptors CD44 and RHAMM may have divergent effects on ozone-induced airway epithelial responses. However, the protective effect of HMWHA is not mediated by either CD44 or RHAMM, but instead by the innate immune receptor TLR5. Our results thus provide new insights into the role of extracellular matrix and specifically HA in ozone-induced lung injury and suggest possible treatment modalities for pollution-induced lung disease.

Ozone concentrations in polluted urban areas can reach levels between 100 and 200 ppb [6], and even up to 400 ppb after industrial accidents or during extreme high pollution days [5,6,7]. In addition, ozone levels are expected to increase in the near future due to increases in ambient temperatures [46]. Therefore, it is essential to understand the pathological mechanisms of ozone-induced lung injury, because of its biological effects on the lung, such as the loss in cell integrity can contribute to lung diseases like asthma and COPD [10].

We compared ozone effects on airway epithelial integrity and transcriptomic responses in human and murine epithelia and saw a striking similarity with significant overlap in gene activation. We found that ozone causes substantial loss of integrity and transcriptional changes in airway epithelia. GSEA revealed a strong upregulation of key inflammatory pathways, including TNFα signaling via NFκB and IL6/JAK/STAT3 signaling. These findings are consistent with the known capacity of ozone to generate danger-associated molecular patterns and activate innate immune signaling pathways [47]. TNFα mediates ozone-induced inflammation in vivo [48] via activation of NFκB and MAPK [49]. We have previously shown that in vivo ozone as well as HA fragments induce a strong NFκB activation in airway epithelial cells [50]. Our current data thus support that epithelial injury and activation of epithelial inflammatory pathways, at least partly via HA fragments, is a central component of ozone-induced lung inflammation. Interestingly, interferon pathways were suppressed in epithelia after ozone exposure, suggesting impaired antiviral defenses that potentially increase susceptibility to viral infections [51,52]. HMWHA reversed these changes, indicating that this treatment may promote immune competence in the airway. We recently found that HMWHA is an effective treatment for severe viral pneumonia in mice and humans [53], which further supports these observations.

Airway remodeling is a crucial feature of chronic lung disease. Notably, TGFβ1 signaling and the epithelial–mesenchymal transition (i.e., EMT) pathway were significantly enriched in ozone-exposed human and murine airway epithelia, supporting that ozone exposure can induce airway remodeling [54], a hallmark of chronic airway diseases such as asthma and COPD [55]. Reactive oxygen species induce TGFβ1 in airway epithelia [56] and HA facilitates TGFβ1-mediated fibrotic responses [57,58]. The concurrent activation of KRAS and MYC target gene sets further supports the notion of a proliferative and stress-adaptive transcriptional response, which may be part of a compensatory repair mechanism but, if prolonged, can drive maladaptive remodeling [59].

Key metabolic pathways, including oxidative phosphorylation, fatty acid metabolism, and cholesterol homeostasis, were significantly downregulated in ozone-exposed airway epithelia (Figure 2A). This may reflect a metabolic shift toward glycolysis, a phenomenon frequently observed in cells undergoing inflammation or stress (Warburg effect) [60] which can promote HA synthesis [61]. We observed an induction in HA secretion by epithelia after ozone exposure, as we also reported after viral infection [53]. HA secretion may thus be a global mechanism of epithelial cells coping with injury. Interestingly, we did not observe a change in HA size with ozone exposure. This may be because ozone does not break down HA disaccharide bonds in a biologically relevant setting, although direct in vitro exposure to both ozone [62] and chlorine gas [63] can degrade HA. Our results may also suggest a possible in vivo coordinated action of cell types in lung injury. We have shown that neutrophils and myeloperoxidase (an oxidative enzyme produced by phagocytes) are necessary for the generation of HA fragments in lung injury [64]. It is therefore possible in injury that structural cells, like epithelia and fibroblasts, produce excess HA which is broken down by immune cells to generate pro-inflammatory HA fragments [65].

HA signaling is mediated by an ever-expanding array of receptors. Our current results suggest an elegant finetuning of HA-mediated injury responses via divergent receptor effects. We found that CD44 and RHAMM deficiency had no effect on airway epithelial integrity after ozone exposure. Forteza et al. has shown that HA fragments induce loss of epithelial integrity after cigarette smoke exposure via the receptor Layilin [66]. Previously we have shown that HMWHA reverses the loss of epithelial integrity induced by HA fragments [64], and we found the same effect now with ozone exposure. Interestingly, however, CD44 and RHAMM again were dispensable for this effect, which was instead mediated by the innate immune receptor TLR5. This finding mirrors previous data by the Noble lab, which found in fibrotic lung injury that HMWHA promotes epithelial integrity via TLR4 [44]. In aggregate these data support that divers HA receptors may promote divergent, sometimes even opposing responses (loss vs. maintenance of epithelial integrity). HA may activate innate immunity without the involvement of prototypic HA receptors like CD44 [67]. Our results support that HMWHA activation of epithelial innate immunity may be a major mechanism of epithelial homeostasis. Interestingly, we and others have previously found that TLR4 and TLR5 can form receptor complexes [43,68], suggesting a possible cooperation of these two receptors in HMWHA signaling as well.

We did find important effects of CD44 and RHAMM deficiency in the transcriptomic response of epithelia to ozone. The most striking observation on a global level was that CD44 deficiency led to a downregulation, while RHAMM deficiency to an upregulation of inflammation and cell activation (see Figure 4). RHAMM deficiency induced pro-inflammatory interleukins 1a and 17a, and suppressed CDKN1A, a potent cell cycle inhibitor, which along with the activation of the cell surface tyrosine kinase ERBB2 (also known as HER2) may explain the activation of cell migration and proliferation-induced pathways also found in the signaling network of these cells. This would surprisingly indicate that epithelial Rhamm is rather a pro-homeostatic, anti-inflammatory factor. Indeed, IL1a is induced in lungs after ozone exposure [48] and in turn induces IL17A. Together, these cytokines recruit inflammatory cells and are associated with steroid-resistant, severe asthma [69]. Although RHAMM is generally viewed as a pro-inflammatory mediator [70], it complexes with the kinase SHP2 [71] which is a negative regulator of inflammation [72,73]. Thus, our results suggest that epithelial RHAMM activation promotes homeostasis and reduces inflammation after ozone exposure. By contrast, epithelial CD44 deficiency was associated with decreased inflammation, cell activation (including ERBB2 suppression) and remodeling pathways (including TGFβ1 suppression) after ozone exposure. This suggests that CD44 is important for the inflammatory response after ozone exposure and supports existing evidence for the role of CD44 in inflammation and remodeling [57,58,74].

HMWHA has strong potential as a treatment agent for chronic lung diseases [18]. We have previously shown beneficial effects in many types of lung injury [75], including translational studies showing its utility in human disease, i.e., COPD exacerbations [76] and COVID-19 [53]. The current results expand the spectrum of HMWHA protective effects to ozone-induced airway epithelial injury. HMWHA effectively mitigated loss of airway epithelial integrity and inflammatory changes, nearly fully reversing the impact of ozone exposure. Notably, 10 of the 19 signaling pathways that were downregulated by HMWHA in both human and mouse cells were previously found to be also induced in common by ozone exposure (compare Figure 2C and Figure 9B), thus supporting that the HMWHA effect on ozone-induced epithelial injury represents a fundamental mechanism. It is intriguing that the protective effects of HMWHA were mediated by TLR5. TLR5 is the cognate receptor for the bacterial protein flagellin [77], but it is increasingly understood that toll-like receptors, including TLR4 and TLR5, can also signal in response to endogenous danger-associated molecules, such as HA [43,44]. We have recently highlighted the beneficial effect of TLR5 in lung injury [31], including promotion of resiliency after bleomycin exposure. We find it extremely intriguing, that both mucosal TLR5 activation [25] and HMWHA expression [78] promote longevity and health span in the murine model. Our current results suggest that this may not be simply a coincidence, but rather that matrix (HMWHA) activation of innate immunity (TLR5) may be a mechanism for sustained organismal fitness. In support of this hypothesis, we found very few qualitative differences in the transcriptomic response of epithelia to HMWHA and a TLR5 agonist (see Supplementary Figure S2), suggesting very similar signaling pathways.

One concern of HMWHA administration is that fragmentation under inflammatory conditions may limit the therapeutic benefit or even exacerbate inflammation. In previous studies, we have shown that therapeutically applied HMWHA escapes clinically relevant degradation. In a clinical study, we treated cystic fibrosis (CF) patients with inhaled HMWHA for 30 days and then evaluated induced sputum HA size and inflammatory cytokines [79]. Although the CF sputum is a highly inflammatory environment, we found that HA size in the sputum increased after HMWHA inhalation. Furthermore, there was no change in sputum inflammatory cytokines before and 30 days after initiation of HMWHA inhalation. In other studies, including human clinical trials with HMWHA nebulization for 5–10 days in patients with significant airway inflammation (COPD exacerbation [76], or severe COVID-19 [53]), we observed a decline in inflammatory markers in human patients and mouse models of lung injury. Therefore, existing evidence supports that HMWHA does not undergo degradation and ameliorates inflammation.

Our work has limitations, including the in vitro investigation of an isolated cell culture model (airway epithelial without immune cells or mesenchymal cells) and a single exposure, whereas real-life conditions always involve combinations of pollutant exposures and cooperation of multiple cells within the tissue and the organism. However, our work enables us to specifically focus on airway epithelial impacts of ozone exposure. We used ozone concentrations that are comparable to real-life exposure conditions, thus increasing confidence that our results carry translational relevance, particularly as we demonstrated strong similarities in the ozone and HMWHA response across species. Furthermore, we investigated HMWHA as a preventative approach, i.e., application prior to oxidative injury, and therefore our results may not apply to HMWHA effects after injury. In previous in vivo work, we have shown that HMWHA given after injury (e.g., chlorine gas exposure [63], viral infection [53], acid instillation [64], and house dust mite exposure [80]) ameliorates adverse phenotypes, i.e., lung inflammation, wet-to-dry lung ratio, histological evidence of lung injury. Although not directly transposable to this study, these results would suggest that therapeutic HMWHA may also have positive effects.

## 5. Conclusions

In summary, our findings demonstrate that in vitro ozone exposure in human and mouse differentiated airway epithelial cell cultures induces significant injury, impairs cell integrity and causes inflammation. HA receptors CD44 and RHAMM have divergent effects on the airway epithelial response to ozone, with RHAMM being protective, while CD44 promotes inflammation. Administration of HMWHA effectively mitigates ozone-induced effects on airway epithelial cells via activation of the innate immune receptors TLR4 and TLR5, thus suggesting that HMWHA may be an effective prophylactic agent for pollution-induced lung injury and in human disease.

## Figures and Tables

**Table 1:** Receptor expression of CD44 and Rhamm pre/post ozone exposure (reads per kilobase million, RPKM) confirmed by RT-PCR (Δ Cq gene minus housekeeping gene 18s). Hmmr = Receptor for hyaluronan-mediated motility. Layn = layilin. Lyve1 = Lymphatic vessel endothelial hyaluronan receptor 1. Stab2 = stabilin 2 (also known as hyaluronan receptor for endocytosis, HARE). Bold = significant change after ozone exposure. NA = not applicable. Note that for RPKM, higher values mean higher gene expression, while for ΔCq, lower values mean higher gene expression.

		Air (Average ± SD)	Ozone (Average ± SD)	*p*-Value Ozone to Air	Air ΔCq (Gene-18s) (Average ± SD)	Ozone ΔCq (Gene-18s) (Average ± SD)	*p*-Value Ozone to Air
**human**	**CD44**	34.48 ± 2.17	42.24 ± 8.18	0.185	5.61 ± 0.24	5.61 ± 0.08	0.9707
	**HMMR**	0.47 ± 0.25	0.34 ± 0.11	0.4597	15.81 ± 0.69	15.35 ± 0.59	0.4322
	**LAYN**	6.09 ± 0.48	7.34 ± 0.56	**0.0425**	8.56 ± 0.22	8.11 ± 0.11	**0.0345**
	**LYVE1**	0	0.00 ± 0.01	0.3739	NA	NA	NA
	**STAB2**	0	0	NA	NA	NA	NA
**mouse**	**Cd44**	46.09 ± 3.45	109.84 ± 13.60	**0.0014**	0.039 ± 0.080	−0.616 ± 0.166	**0.0035**
	**Hmmr**	1.83 ± 0.35	1.39 ± 0.17	0.1231	8.26 ± 0.06	9.24 ± 0.12	**0.0003**
	**Layn**	0.37 ± 0.03	0.15 ± 0.03	**0.0007**	6.64 ± 0.40	6.87 ± 0.31	0.4690
	**Lyve1**	0.04 ± 0.07	0	0.3739	NA	NA	NA
	**Stab2**	0.01 ± 0.0.01	0.01 ± 0.0.01	0.7489	NA	NA	NA

## Data Availability

Transcriptomic data described in this study will be deposited in GEO. All other raw data are available by the corresponding author upon written request.

## Supplementary Materials

The following supporting information can be downloaded at: https://www.mdpi.com/article/10.3390/biom16060795/s1, Figure S1: No change in TEER in genetically Cd44- or Hmmr-deficient cells, compared to wildtype (C57Bl/6J) cells; Figure S2: Ingenuity Pathway Analysis of human ALI DEGs in response to either TLR5 agonist or HMWHA exposure; Supplemental Methods.
