# Supplementary material for: Hyaluronan Signaling Ameliorates the Epithelial Injury Response and Barrier Disruption After Ozone Exposure
Source: Biomolecules. 2026 May 28;16(6):795. doi: 10.3390/biom16060795 (PMC13296466; doi:10.3390/biom16060795)
Supplement: Supplementary file 1 [file biomolecules-16-00795-s001.zip › biomolecules-4165441-supplementary.pdf]

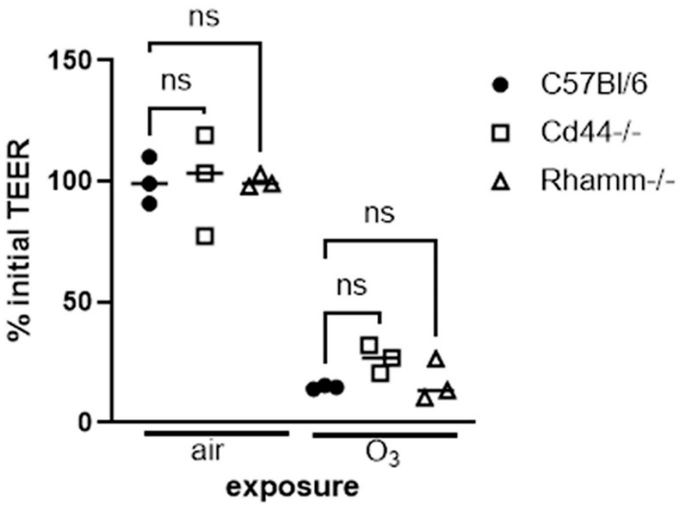

Figure S1. No change in TEER in genetically Cd44- or Hmmr-deficient cells, compared to wildtype (C57Bl/6J) cells. ns=nonsignificant, ANOVA with multiple comparison analysis.

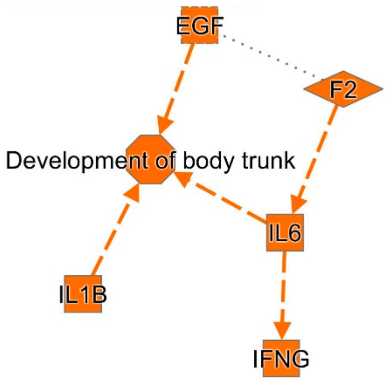

Figure S2. Ingenuity Pathway Analysis of human ALI DEGs in response to either TLR5 agonist or HMWHA exposure. EGF=epithelial growth factor. IL6=Interleukin 6. IL1B=Interleukin 1 β, IFNG = Interferon γ. F2= prothrombin. The relative upregulation of these pathways may simply reflect a stronger activity of the TLR5 agonist compared to HMWHA, as they are downstream of the TLR5 signaling pathway.

Supplemental Methods

Mouse ALI media

Medium	Component	Provider (catalogue number)	Final concentration
MTEC Basic (*)	DMEM/F12	Gibco (1133032)	
	Penicillin<br>Streptomycin	Lonza (DE17-602e)	100U/ml<br>100µg/ml
	NaHCO3	Gibco (25080094)	0.03% (w/v)
MTEC proliferation medium	MTEC basic	(*)	
	L-Glutamine	Gibco (25030081)	1.5mM
	Fetal Calf Serum	HyClone (SH30071.03)	5%

 https://doi.org/10.3390/biom16060795

	ITS-G	Gibco (41400045)	1x
	Cholera Toxin	Sigma (C8052-0,5mg)	0.1μg/ml
	Murine EGF	Peprotech (315-09)	0.025μg/ml
	Bovine Pituitary Extract	Gibco (13028014)	0.03mg/ml
	Y-27632 (add fresh)	Cayman Chemical (10005583)	10μM
	Retinoic Acid	Sigma (R625-50mg)	0.05μM
MTEC differentiation medium	MTEC basic		
	L-Glutmine	Gibco (25030081)	1.5mM
	Bovine Serum Albumin	Gibco (15260037)	0.1% (w/v)
	ITS-G	Gibco (41400045)	1x
	Cholera Toxin	Sigma (C8052-0,5mg)	0.1μg/ml
	Murine EGF	Peprotech (315-09)	0.025μg/ml
	Bovine Pituitary Extract	Gibco (13028014)	0.03mg/ml
	Y-27632 (add fresh)	Cayman Chemical (10005583)	10μM
	Retinoic Acid	Sigma (R625-50mg)	0.05μM
KSFM expansion medium	KSFM	Gibco (17005034)	
	Penicillin<br>Streptomycin	Lonza (DE17-602e)	100U/ml<br>100μg/ml
	Murine EGF	Peprotech (315-09)	0.025μg/ml
	Bovine Pituitary Extract	Gibco (13028014)	0.03mg/ml
	Isoproterenol	Sigma (I-6504)	1μM
	Y-27632 (add fresh)	Cayman Chemical (10005583)	10μM
	DAPT (add fresh)	Sigma (D5942)	5μM

Mouse ALI other reagents for digestion

Product	Provider
Rat tail collagen (#354236)	Corning, MA, USA
Glacial Acid	Sigma Aldrich, USA
Transwell permeable supports 12mm Insert, 12 well plate, 0.4μm Polyester membrane	Costar (#3460)
Corning primaria	Corning (#353801)
Pronase 1g from Streptomyces griseus, 10165921001	Roche Diagnostics, Mannheim, Germany
10mg DNase	Sigma (#DN25)
Cell strainer	Corning (#352350)

Human ALI media

PneumaCult ALI Basal medium #05002	StemCell Technologies, USA
PneumaCult ALI 10X Supplement #05003	StemCell Technologies, USA
PneumaCult ALI Maintenance Supplement #05006	StemCell Technologies, USA
Animal Component-Free Cell Dissociation Kit	StemCell Technologies, USA

• ACFEnzymaticDissociationSolution<br>• ACFEnzymeInhibitionSolution #05426	
Heparin Solution #07980	StemCell Technologies, USA
Hydrocortisone Stock Solution #07925	StemCell Technologies, USA
PneumaCult Ex Plus Medium #05040	StemCell Technologies, USA

Kits

Product	Provider
RNeasy mini kit (250) (#74106)	Qiagen, USA
DuoSet ELISA Development System Hyaluronan	R&D Systems, Inc. (#DY3614-05) 614 McKinley Place NE, Minneapolis, MN 55413, USA
Applied Biosystems; High-Capacity cDNA Reverse Transcriptase Kit	Thermo Fisher Scientific Baltics, UAB
Stranded mRNA Prep Kit	Illumina, USA

qRT-PCR Primers

Product	Sequence	Provider
Mouse RM2_IL6	5′TGCATCATCGTTGTTCATAC	Sigma-Aldrich
Mouse FM2_IL6	5′GTCTATACCACTTCACAAGTC	Sigma-Aldrich
Mouse 18s For(1)	5′TACCTGGTTGATCCTGCCAG	IDT
Mouse 18s Rev(1)	5′CCGTCGGCATGTATTAGCTC	IDT
Mouse FM1_CXCL1	5′AAAGATGCTAAAAGGTGTCC	Sigma-Aldrich
Mouse BM1_CXCL1	5′GTATAGTGTTGTCAGAAGCC	Sigma-Aldrich
Mouse FM1_CXCL2	5′GGGTTGACTTCAAGAACATC	Sigma-Aldrich
Mouse BM1_CXCL2	5′CCTTGCCTTTGTTCAGTATC	Sigma-Aldrich

Instruments

Product	Provider
Millicell ERS-2 Electrical Resistance System	EMD Millipore Corporation Burlington, MA U.S.A., an affiliate of Merck KGaA, Darmstadt, Germany
BioTek Power Wave XS (ELISA)	BioTek, USA
Qubit 4 Flex Fluorometer	Thermo Fisher Scientific, USA
Tape Station 4200	Agilent, USA
BIO RAD CFX Connect Real-Time System (PCR)	BIO RAD, USA
GeneAmp PCR System 9700	Applied BioSystems?
Peltier Thermal Cycler, DNA engine	BIORAD, USA
Forma seris3 water jacketed C02 incubator Model # 4130	Thermo Fisher Scientific, USA
DAIGGER Vortex Genie 2 (#3030A)	DAIGGER Scientific
Heraeus Multifuge X1R	Thermo Fisher Scientific, USA
Pipette Controler SP+	Mettler Toledo, OH, USA
Evos XL Core Imaging System	Thermo Fisher Scientific, USA
Rainin Pipet Lite XLS SL-10, 20, 200, 1000	Mettler Toledo, USA
Rainin Pipet Lite XLS L-50	Mettler Toledo, USA

https://doi.org/10.3390/biom16060795

BIO RAD TC20 Automated Cell Counter	BIO RAD, USA
Zeiss LSM880	Carl Zeiss Inc, Oberkochen, Germany
Zeiss LSM 780 inverted confocal microscope	Carl Zeiss, Inc, Oberkochen, Germany

Cells and mice

**Product**	**Provider**
Human primary bronchial epithelial cells	Lonza, Rockville, USA
MatTek human cells	MatTek, Ashland, MA, USA
C57BL/6J Jax 000664	NIEHS internal breed
B6.129(Cg)-Cd44<tm1Hbg>/J Jax 005085	The Jackson laboratory, Bar Harbor, ME, USA
B6.129P2-Hmmr<tm1Baa>	NIEHS colony
B6.129P2(SJL)-Myd88<tm1.1Defr>/J Jax 9088	The Jackson laboratory, Bar Harbor, ME, USA

Chemicals and Reagents

**Trypan Blue #07050**	**StemCell Technologies, Vancouver, Canada**
Gibco Phosphate Buffered Saline	Thermo Fisher Scientific, USA
Ethyl Alkohol	The Warner Graham Company, Maryland, USA
Ultra-Pure DEPC Treated Water	Invitrogen by life technologies, CA, USA
CO2	
Power SYBR Green PCR Master Mix	Applied biosystems by Thermo Fisher Scientific, Warrington, UK
DNA Digest Buffer RDD	Qiagen, USA
RNAse free water	Qiagen USA
RNA Screen Tape	Agilent Technologies, USA
RNA Screen Tape Sample Buffer	Agilent Technologies, USA
Wash Buffer #WA126	R&D Systems, MN, USA
Reagent Diluent #DY004	R&D Systems, MN, USA
Substrate Solution #DY999	R&D Systems, MN, USA
Stop Solution #DY994	R&D Systems, MN, USA
DAPI (4′6′-diamidino-2-phenylindole) #62248	Thermo Fisher Scientific, USA
Prolong Diamond Antifade Mountant # P36961	Thermo Fisher Scientific, USA
Triton X-100 # X100-100ML	Millipore Sigma, USA
Normal Donkey Serum #017-000-121	Jackson Immunoresearch, USA
Paraformaldehyde	Sigma – Aldrich, USA
Tris buffered Saline	Sigma – Aldrich, USA

Consumables

**Product**	**Provider**
Costar 3460 12mm Insert, 0.4µm Polyester membrane	Costar, ME, USA
Hard-Shell PCR Plates 96 Well, thin wall (#HSP9601)	BIO-RAD
Falcon Tubes	Corning Science Mexico S.A. de C.V.
Pipet Tips with filter 10µl, 20µl, 200µl, 1250µl	VWR Radnor, PA, USA

https://doi.org/10.3390/biom16060795

Reaction tubes 1,5ml / 2ml / 5ml	Eppendorf North America, USA
TC Flask T75	Thermo Fisher Scientific, USA
10ml Syringe	Becton Dickinson, NJ, USA
Corning Primaria 353801	Corning, NC, USA
Dual Chamber Cell Counting Slides #1450011	BIO RAD, USA
Petri dish Falcon	Corning incorporated-life sciences, NC, USA
Glas pipettes 10ml, 25ml	Falcon, NC, USA
50ml tube	Falcon, NC, USA
15ml tube	Sarstedt, NC, USA
Plate Sealer #DY992	R&D Systems, MN, USA

2.1.10. Antibodies

**Product**	**Provider**
Mouse anti-ZO-1 #33-9100	Thermo Fisher Scientific, USA
Donkey anti-mouse IgG, Alexa Fluor 594 # A21203	Thermo Fisher Scientific, USA

Software

**Product**	**Provider**
CFX Maestro Software (PCR)	BIO RAD, USA
GraphPad Prism Version 10.2.3 for MacOS	GraphPad Software, Inc.; Boston, USA
Mendeley	Elsevier, Amsterdam
Applied Biosystems QuantStudio Software	ThermoFisher Scientific, USA
TapeStation Controller Software 4.1.1	Agilent, USA
Trim Galore 0.6.10	Babraham Bioinformatics
STAR splice-aware aligner 2.6.0c	Cold Spring Harbor Laboratory, NY, USA
featureCounts 2.0.6	Walter and Eliza Hall Institute of Medical Research, Melbourne, Australia
edgeR package 4.0.16	Walter and Eliza Hall Institute of Medical Research, Melbourne, Australia
Ingenuity Pathway Analysis	Qiagen, USA
Gene Set Enrichment Analysis 4.3.2	Broad Institute of MIT and Harvard, MA, USA
Molecular Signatures Databases MSigDB 2024.1.Mm	Broad Institute of MIT and Harvard, MA, USA
Molecular Signatures Databases MSigDB 2024.1.Hs	Broad Institute of MIT and Harvard, MA, USA
FIJI 1.54f	National Institutes of Health, Bethesda, USA
labkit	Max Planck Institute, Dresden, Germany
Imaris 9.9	Oxford Instruments plc, Abington UK
Zen microscopy software	Carl Zeiss Microscopy GmbH, Jena, Germany

https://doi.org/10.3390/biom16060795
